# Mono-HOPE Versus Dual-HOPE in Liver Transplantation: A Propensity Score-Matched Evaluation of Early Graft Outcome

**DOI:** 10.3389/ti.2025.13891

**Published:** 2025-02-05

**Authors:** Dominik Thomas Koch, Micol Tamai, Malte Schirren, Moritz Drefs, Severin Jacobi, Christian M. Lange, Matthias Ilmer, Hanno Nieß, Bernhard Renz, Jens Werner, Markus Guba, Dionysios Koliogiannis

**Affiliations:** ^1^ Department of General, Visceral and Transplantation Surgery, LMU University Hospital, LMU Munich, Munich, Germany; ^2^ Transplantation Center Munich, LMU University Hospital, LMU Munich, Munich, Germany; ^3^ Department of Internal Medicine II, LMU University Hospital, LMU Munich, Munich, Germany; ^4^ Division of General Surgery, Mayo Clinic, Rochester, MN, United States

**Keywords:** liver transplantation, end-ischemic liver preservation, hypothermic oxygenated machine perfusion, mono-Hope, dual-HOPE

## Abstract

Hypothermic oxygenated machine perfusion (HOPE) has become an integral technique to enhance donor graft function in liver transplantation (LiTx). This study compares early posttransplant outcomes of mono-HOPE (portal vein perfusion only) versus dual- HOPE (both portal vein and hepatic artery perfusion). A retrospective analysis was conducted on 183 LiTx recipients, with 90 receiving mono-HOPE and 93 receiving dual-HOPE grafts. Propensity Score Matching (PSM) was applied, resulting in a matched cohort of 146 patients. Primary outcomes included one-year patient and graft survival, and non-anastomotic biliary strictures (NAS). Secondary outcomes included hospital length of stay (HLS). One-year patient survival was 81.7% in the mono-HOPE and 81.7% in the dual-HOPE group, and overall survival did not differ (p = 0.990). One-year death-censored graft survival was similarly comparable (91.2% vs. 93.3%, p = 0.893). NAS were observed in 10.96% in the mono-HOPE and 8.22% in the dual-HOPE group (p = 0.574). The median HLS was 29 days for both groups. Results suggest that dual-HOPE did not significantly improve patient or graft survival, nor did it reduce NAS or HLS compared to mono-HOPE. Assuming that larger cohorts and long-term follow-up data confirm this, additional cannulation of the hepatic artery during machine perfusion in hypothermic conditions may not be beneficial.

## Graphical Abstract

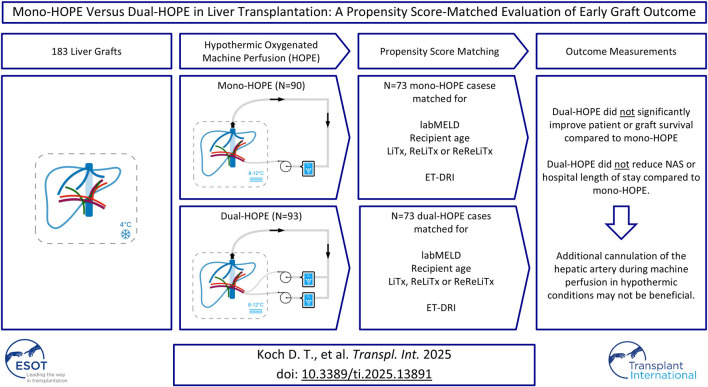

## Introduction

Liver transplantation (LiTx) remains the definitive therapeutic option for patients suffering from end-stage liver diseases and for selected liver malignancies, significantly improving patient survival and quality of life. However, the scarcity of suitable donor grafts has driven the use of extended criteria donors (ECD) to expand the donor pool. These grafts, though increasing the availability of organs, are associated with a higher risk of complications, including delayed graft function (DGF) and biliary complications (BC).

Hypothermic oxygenated machine perfusion (HOPE) has emerged as a valuable technique in preserving ECD livers. By supplying oxygenated perfusate at low temperatures, HOPE minimizes ischemia-reperfusion injury (IRI) – a key factor contributing to graft dysfunction and failure – by providing oxygen and nutrients to the graft at low temperatures, thereby maintaining metabolic activity at a reduced rate and minimizing the accumulation of reactive oxygen species (ROS) [1–3]. Most notably, HOPE has been shown to improve early graft function and reduce BC, which are more prevalent in ECD grafts. HOPE improves electrolyte balance, enhances hemodynamic stability, lowers post-reperfusion syndrome (PRS) incidences, and shows overall improved outcomes [4–6]. It has gained considerable traction in recent years due to its relatively simple initiation process and its cost-effectiveness, reflected in shorter intensive care unit (ICU) and hospital length of stay [7]. As a result, HOPE is increasingly being used, not only in ECD livers.

HOPE can be administered in two configurations: Perfusion of the portal vein only (mono-HOPE) and perfusion of both the portal vein and the hepatic artery (dual-HOPE). The dual-HOPE approach is hypothesized to provide superior graft protection by delivering oxygenated perfusate through both vascular systems, thereby enhancing the preservation of the entire liver parenchyma and, crucially, the bile ducts. Dual vascular perfusion is believed to mitigate the risk of ischemic cholangiopathy, a severe and potentially life-threatening complication that can occur following transplantation.

Despite the hypothesized benefits, limited comparative studies exist regarding the efficacy of dual-HOPE versus mono-HOPE in liver graft preservation. This study aims to elucidate the impact of these two perfusion modalities on early post-transplant outcomes through a Propensity Score-matched analysis, focusing on metrics such as patient survival, graft survival, non-anastomotic biliary strictures (NAS), and hospital length of stay (HLS).

## Patients and Methods

### Study Design

This is a single-center, retrospective cohort study conducted at the Department of General, Visceral, and Transplant Surgery, LMU University Hospital Munich, Germany. The study’s primary objective was to compare the outcome of LiTx recipients who received grafts preserved using end-ischemic mono-HOPE versus dual-HOPE from October 2019 to May 2024.

### Study Population

In this analysis, we included 183 patients who underwent orthotopic liver transplantation during the study period. Patients were included based on the following criteria:

#### Inclusion Criteria

- Adult patients (≥18 years old) who received liver grafts preserved with end-ischemic HOPE.
- Availability of complete clinical and follow-up data.
- The received grafts were preserved in Histidine-tryptophan-ketoglutarate solution (HTK) and transported on ice.
- Only DBD (Donation after brain death) organs were included, as there is no DCD (Donation after cardiac death) program in Germany.

#### Exclusion Criteria

- Patients who underwent combined organ transplantation, e.g., liver and kidney or liver and lung, were excluded to avoid confounding factors.
- Patients who received grafts that were preserved by static cold storage (SCS) only.

Regardless of surgeon preferences, all grafts fulfilling these criteria were allocated to end-ischemic HOPE. There was no distinction between extended criteria donor (ECD) and non-ECD grafts. Among the included patients, 90 received grafts preserved by end-ischemic mono-HOPE, and 93 received grafts preserved by end-ischemic dual-HOPE.

### HOPE Protocol

All liver grafts were preserved and transported to our center using the standard protocol of SCS in HTK solution on ice. Upon arrival, grafts were prepared for implantation at the back table. This also included artery dissections and any necessary arterial reconstructions. Following back table preparation, the subsequent protocols for HOPE were conducted:

#### Mono-HOPE Protocol

- Only the portal vein was cannulated for machine perfusion.
- Machine perfusion was initiated using the LiverAssist^®^ device (XVIVO, Groningen, Netherlands, and Göteborg, Sweden) with University of Wisconsin machine perfusion solution (UW-MPS) maintained at a temperature of 8°C–12°C.
- Portal vein pressure was adjusted to 3–5 mmHg, with continuous monitoring of flow rates, aiming for a flow rate of 100–150 mL/min [6].
- Machine perfusion was conducted until the recipient wasready for graft implantation.

#### Dual-HOPE Protocol

- Both the portal vein and the hepatic artery were cannulated for perfusion.
- Machine perfusion was initiated with the LiverAssist^®^ device, and UW-MPS was maintained at a temperature of 8°C–12°C as for mono-HOPE.
- Perfusion of the portal vein followed the same protocol as for mono-HOPE.
- Hepatic artery pressure was adjusted to 20–25 mmHg, with continuous monitoring of flow rates.
- Machine perfusion was conducted until the recipient wasready for graft implantation.

In both protocols, perfusion was continued throughout the recipients’ hepatectomy. Immediately before implantation, the grafts were flushed with HTK solution to remove residual UW-MPS.

Our approach to machine perfusion techniques evolved as we gained experience, resulting in two different eras regarding the criteria used to determine which perfusion technique was performed. Initially, we focused exclusively on mono-HOPE to develop confidence and refine our expertise with the method. As our proficiency increased, we transitioned to dual-HOPE whenever cannulation of the hepatic artery was feasible. This progression highlights the deliberate, stepwise approach we adopted to ensure safety and effectiveness. However, there was no randomization.

### Liver Transplantation and Immunosuppression

LiTx was predominantly performed using the vena cava-preserving “piggyback” technique. Bile duct reconstruction was achieved through duct-to-duct anastomosis when feasible. Immunosuppression was initiated with a standard triple therapy regimen of tacrolimus, mycophenolate mofetil, and corticosteroids. Corticosteroids were tapered and discontinued by the third post-transplant month, with tacrolimus further reduced, except in cases requiring prolonged use due to clinical indications such as acute rejection or autoimmune hepatitis (AIH).

In patients transplanted for malignancy, immunosuppression was modified at 3 months by transitioning from mycophenolate to an mTOR inhibitor, such as Everolimus, in combination with low-dose tacrolimus. This strategy aims to reduce the risk of tumor recurrence while preserving effective graft protection [8, 9].

Additionally, patients received standard anti-infective prophylaxis for 6 months, including sulfamethoxazole-trimethoprim and valganciclovir.

### Outcome Measures

Multiple parameters were analyzed. Primary outcomes included patient survival, graft survival, and incidence of non-anastomotic biliary strictures (NAS) within the first year. NAS were defined as one or more focal areas of narrowing of the bile ducts proximal to the biliary anastomosis [10–12]. Magnetic resonance cholangiopancreatography (MRCP) and direct cholangiography through endoscopic retrograde cholangiography (ERC) and percutaneous cholangiography (PTC) for diagnosis of NAS were obtained based on clinical indications.

Secondary outcomes included hospital length of stay (HLS) following LiTx.

Further data were collected to characterize the study population and to perform Propensity Score Matching (PSM).

All patient data were extracted from our institutional electronic medical records system, and relevant clinical information was reviewed by two independent investigators to ensure accuracy.

### Propensity Score Matching

**FIGURE 1:**
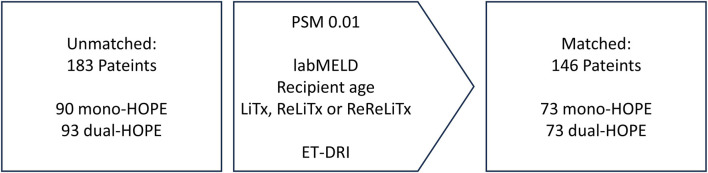
Propensity score matching; labMELD, lab Model for End-stage Liver Disease; LiTx, liver transplantation; ReLiTx, Re-liver ransplantation; ReReLiTx, ReRe-liver Transplantation; ET-DRI, Eurotransplant-Donor Risk Index.

To reduce the impact of potential confounders and ensure comparability between the mono-HOPE and dual-HOPE group, Propensity Score Matching (PSM) was applied. The following recipient variables were used for matching: lab Model for End-stage Liver Disease (labMELD) score, recipient age, and whether the LiTx was the first, second, or third for the patient. The Eurotransplant-Donor Risk Index (ET-DRI) was utilized as a representative donor variable. There were no missing data, and a Propensity Score match tolerance of 0.01 with a 1-to-1 matching method was applied, resulting in a matched cohort of 146 patients (73 patients in each group) for further analysis (Figure 1).

The ET-DRI includes donor age, cause of death (COD), donation after cardiac death (DCD) or donation after brain death (DBD), partial/split or whole liver, regional or national share, cold ischemia time, latest GGT and if it was a rescue offer:

ET-DRI = exp{0.960 × [(0.154 if 40≤age<50) + (0.274 If 50≤age<60) + (0.424 if 60≤age<70) + (0.501 if 70≤age) + (0.079 if COD = anoxia) + (0.145× if COD = cerebrovascular accident) + (0.184 if COD = other) + (0.411 if DCD) + (0.422 if partial/split) + (0.105 if regional share) + (0.244 if national share)] + [0.010 × (cold ischemia time−8 h)] + 0.06 × {[latest lab GGT (U/L) - 50]/100} + (0.180 if rescue offer)}.

PSM was performed using IBM SPSS Statistics 29 (IBM, Armonk, New York, United States).

### Statistical Analysis

All statistical analyses were performed using IBM SPSS Statistics 29 (IBM, Armonk, New York, United States).

The Propensity Score-matched data were treated as a regular dataset, and conventional statistical analyses were applied. Therefore, the matched data were regarded as pooled data, and the analyses were conducted under the independence assumption:

- Continuous variables were compared using unpaired t-tests for normally distributed data. Welch’s correction was applied when variances were unequal (as determined by a Levene test with a p < 0.05).
- Cohen’s d was used to calculate standardized mean differences (SMD).
- Categorical variables were compared using the Chi-square-test.
- Survival analyses and time-to-event analyses were performed using Kaplan-Meier curves, and comparisons between the two groups were made using the log-rank test.
- Graft survival was assessed by non-death-censored and death-censored graft survival. For non-death-censored graft survival, the graft was considered as non-functioning when the patient died. Therefore, an event was defined as Re-liver transplantation (ReLiTx) or death. For death-censored graft survival, an event was only defined as ReLiTx, and death was censored.
- A p-value of less than 0.05 was considered statistically significant.

## Results

### Patient Characteristics of the Propensity Score-Matched Study Population

**FIGURE 2:**
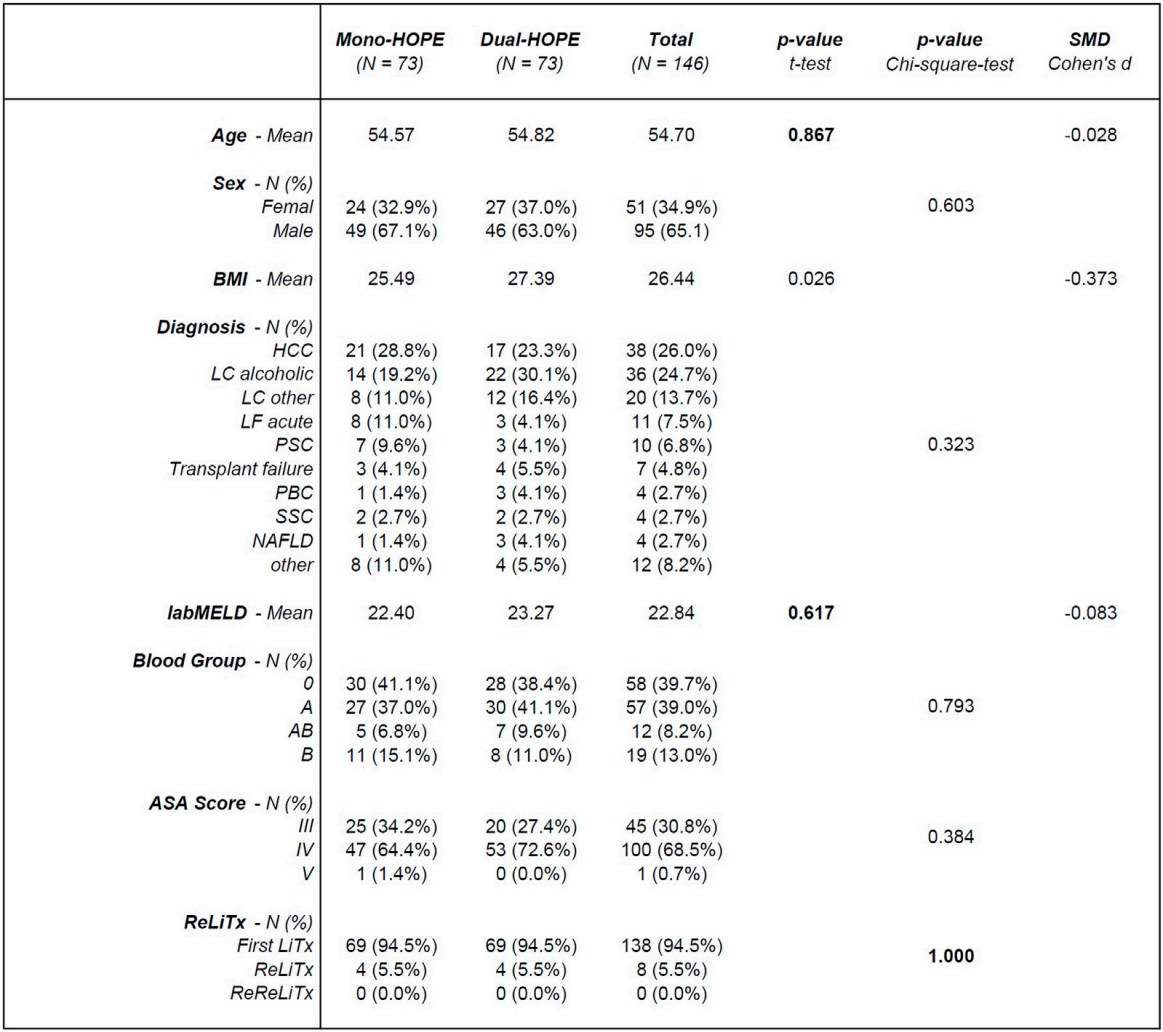
Patient characteristics; SMD, standardized mean difference; BMI, body mass index; HCC, hepatocellular carcinoma; LC, liver cirrhosis; LF, liver failure; PSC, primary sclerosing cholangitis; PBC, primary biliary cholangitis; SCC, secondary sclerosing cholangitis; NAFLD, non-alcoholic fatty liver disease; labMELD, lab Model for End-stage Liver Disease; ASA Score, American Society of Anesthesiologists Score; LiTx, liver transplantation; ReLiTx, Re-liver transplantation; ReReLiTx, ReRe-liver transplantation.

The PSM resulted in a matched cohort of 146 patients, with 73 patients in both the mono- and the dual-HOPE group. The patient characteristics are shown in Figure 2.

There was no significant difference in the patient-associated variables used for PSM, confirming a successful matching process: Age (p = 0.867), labMELD (p = 0.617), and whether the liver transplantation was the first, second, or third for the patient (p = 1.000).

Except for the body mass index (BMI) with a p-value of 0.026, all other patient characteristics did not differ significantly between the two groups: Sex (p = 0.603), diagnosis (p = 0.323), blood group (p = 0.793) and ASA Score (p = 0.384).

### Donor Characteristics and Machine Perfusion Times of the Propensity Score-Matched Study Population

**FIGURE 3:**
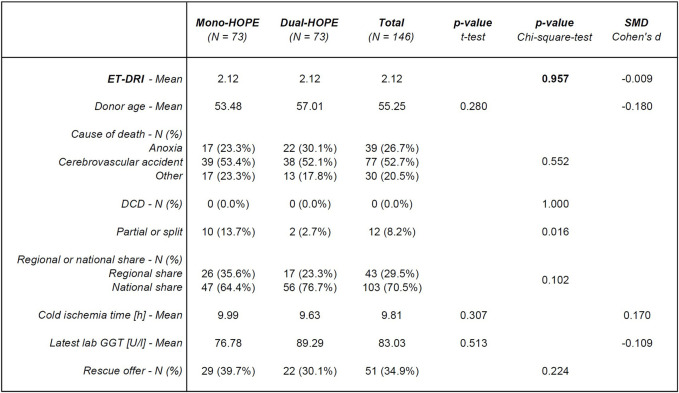
Donor characteristics; SMD, standardized mean difference; ET-DRI, Eurotransplant-Donor Risk Index; DCD, Donation after cardiac death; lab GGT, lab Gamma-Glutamyl-Transferase.

The donor characteristics for the Propensity Score-matched cohort of 146 patients are shown in Figure 3. There was no significant difference in the donor-associated variable ET-DRI used for PSM, confirming a successful matching process (p = 0.957). Germany has no DCD program, so no DCD organs were included. Except for the number of partial or split liver grafts with a p-value of 0.016, all other donor characteristics did not differ significantly between the two groups: Donor age (p = 0.280), cause of death (p = 0.552), regional or national share (p = 0.102), cold ischemia time (p = 0.307), latest lab GGT (p = 0.513), and number of rescue offers (p = 0.224).

The Propensity Score-matched mono-HOPE group’s mean machine perfusion time was 154.15 min, ranging from 35.0 min to 370.0 min. The mean machine perfusion time in the dual-HOPE group was 178.94 min, ranging from 60.0 min to 480.0 min. With a p-value of 0.097 for the unpaired t-test, the difference was not significant.

### Patient Survival

**FIGURE 4:**
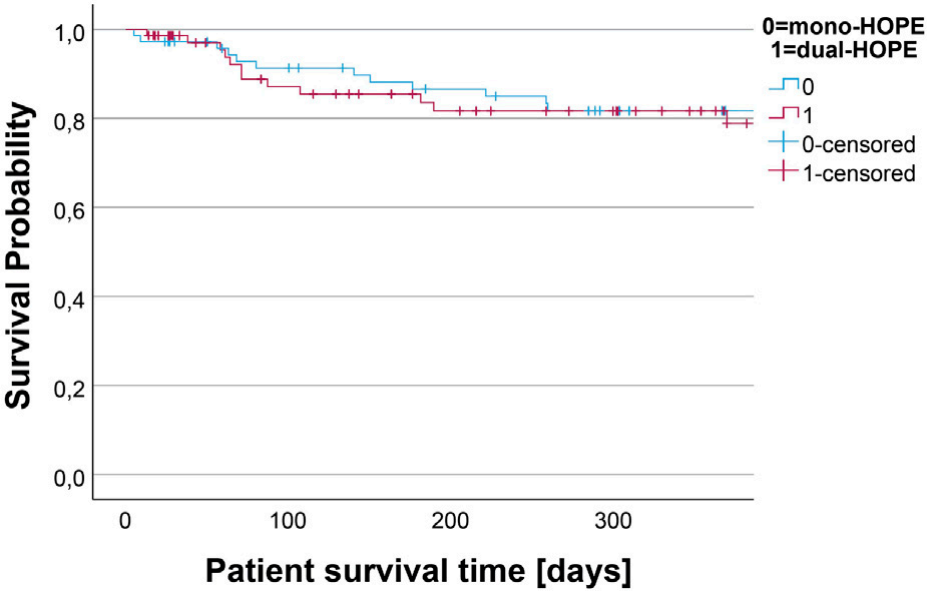
Kaplan-Meier curve for patient survival.

Patient survival was assessed by Kaplan-Meier analysis. There was no difference in the survival curves of the mono- and dual-HOPE group (log-rank: p = 0.990). The median follow-up time assessed with reverse Kaplan-Meier was 872 days for the mono-HOPE group and 367 days for the dual-HOPE group. The overall one-year patient survival was 81.7% (SD 4.8%) in the mono-HOPE group and 81.7% (SD 5.0%) in the dual-HOPE group. The Kaplan-Meier curves for patient survival are shown in Figure 4.

### Graft Survival

ReLiTx had to be performed in 6 out of 73 (8.22%) cases in the mono-HOPE group and 5 out of 73 (6.85%) cases in the dual-HOPE group. The Chi-square-test revealed no significant difference between the two groups (p = 0.754).

The graft survival was assessed by Kaplan-Meier analysis. We assessed non-death-censored and death-censored graft survival.

**FIGURE 5:**
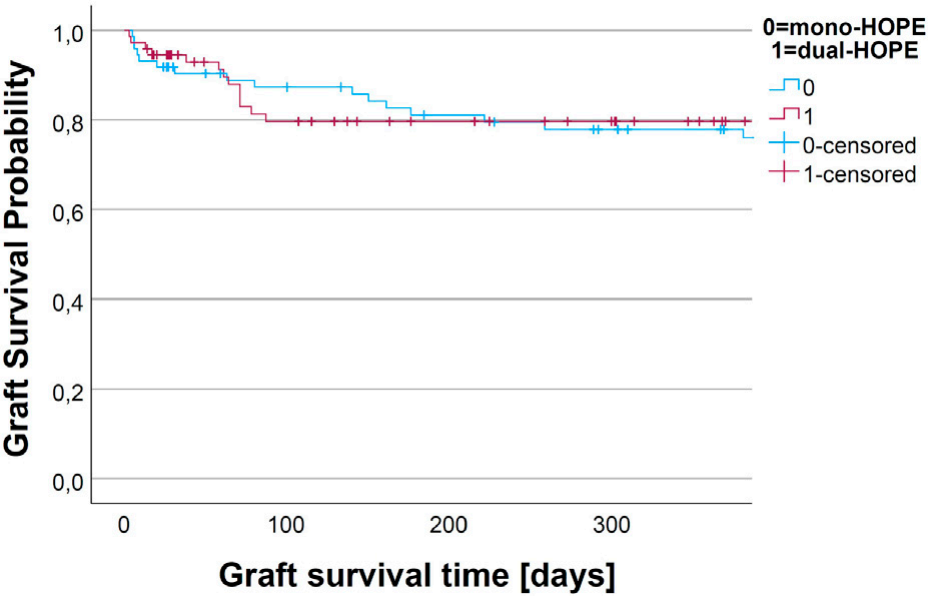
Kaplan-Meier curve for non-death-censored graft survival.

For non-death-censored graft survival, the graft was considered as non-functioning when the patient died. There was no difference in the non-death-censored graft survival curves of the mono- and dual-HOPE group (log-rank: p = 0.899). The one-year non-death-censored graft survival was 77.9% (SD 5.1%) in the mono-HOPE group and 79.6% (SD 5.1%) in the dual-HOPE group. The Kaplan-Meier curves for non-death censored graft survival are shown in Figure 5.

**FIGURE 6:**
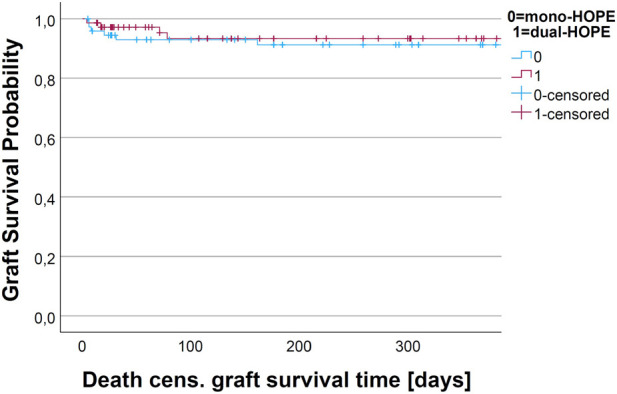
Kaplan-Meier curve for death-censored graft survival.

There was no difference in the death-censored graft survival curves of the mono- and dual-HOPE group (log-rank: p = 0.893). The one-year death-censored graft survival was 91.2% (SD 3.4%) in the mono-HOPE group and 93.3% (SD 3.3%) in the dual-HOPE group. The Kaplan-Meier curves for death-censored graft survival are shown in Figure 6.

### Non-Anastomotic Biliary Strictures

All NAS detected with MRI, ERC, or PTC within 1 year were recorded. There were no protocol MRI scans conducted.

NAS occurred in 8 out of 73 (10.96%) cases in the mono-HOPE group and 6 out of 73 (8.22%) cases in the dual-HOPE group within the first year following LiTx. This difference did not reach statistical significance (Chi-square-test: p = 0.574). With an inclusion time from October 2019 to May 2024, not all patients reached a full follow-up of 1 year.

### Hospital Length of Stay

Cumulative ICU days were assessed for all patients, including patients who died during the hospital stay. Patients in the mono-HOPE group had a median of 6 cumulative ICU days (mean 22.41 days), and patients in the dual-HOPE group had a median of 7 cumulative ICU days (mean 14.84 days). The t-test with Welch’s correction was used as the Levene test was significant (Leven test: p = 0.001). The t-test with Welch’s correction did not show a difference between the two groups (p = 0.110).

Hospital length of stay (HLS) was analyzed by a time-to-event analysis. Patients who died were censored. The median HLS in both groups was 29 days and there was no significant difference in the two time-to-event curves of the two groups (log-rank: p = 0.331).

### Sensitivity Analysis Excluding Recipients of Partial and Re-Liver Transplantations

**FIGURE 7:**
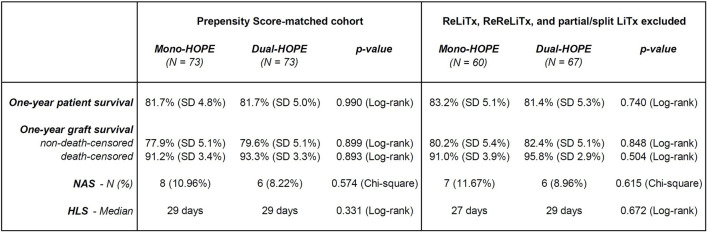
Sensitivity analysis excluding recipients of partial and Re-liver transplantations; Original Propensity Score-matched cohort (left) and subgroup with recipients of ReLiTx, ReReLiTx, and partial or split LiTx excluded (right); LiTx, liver transplantation; ReLiTx, Re-liver transplantation; ReReLiTx, ReRe-liver transplantation; SD, standard deviation; NAS, non-anastomotic biliary strictures; HLS, hospital length of stay.

Recipients of ReLiTx, ReReLiTx, and partial or split LiTx were excluded from the Propensity Score-matched cohort to perform a sensitivity analysis. The sensitivity analysis showed comparable results to the original Propensity Score-matched cohort. There were still no significant differences regarding one-year patient survival, one-year graft survival, NAS, and HLS between the mono- and dual-HOPE group (Figure 7).

## Discussion

End-ischemic hypothermic oxygenated machine perfusion (HOPE) has become an increasingly valuable technique for graft preconditioning prior to liver transplantation (LiTx), particularly for extended criteria donor (ECD) grafts, which are more susceptible to ischemia-reperfusion injury (IRI) [13–15]. Previous studies have demonstrated that HOPE can effectively mitigate this injury, resulting in improved early graft function and reduced incidence of cholangiopathy, notably non-anastomotic biliary strictures (NAS) within the first year post-transplantation – a significant complication associated with ECD liver grafts [1–3].

Our study evaluated the impact of dual-HOPE, which perfuses both the portal vein and the hepatic artery, compared to the standard mono-HOPE, which only perfuses the portal vein.

Our findings did not show a statistically significant improvement in patient or graft survival with dual-HOPE compared to mono-HOPE. These results suggest that adding hepatic artery perfusion may not confer additional protection to the graft. This finding contrasts with the initial hypothesis that dual vascular perfusion would offer enhanced preservation of the entire hepatic parenchyma and, by extension, improve early graft function and survival.

While the dual-HOPE group exhibited a lower incidence of NAS compared to the mono-HOPE group, this difference did not reach statistical significance. In addition, the shorter follow-up time of the dual-HOPE group could be an explanation for falsely reduced NAS incidence in this group. The original hypothesis – that dual-HOPE would provide superior graft protection by delivering oxygenated perfusate through both vascular systems, thereby enhancing the preservation of the entire liver parenchyma and, crucially, the bile ducts – must, therefore, be reconsidered. The lack of significant impact on NAS incidence indicates that the theoretical advantages of dual vascular perfusion do not translate into measurable clinical benefits, at least in the context of our study population.

After Propensity Score Matching, the mono-HOPE and dual-HOPE group were well-balanced, with the only significant difference in donor characteristics being BMI and the amount of partial or split organs. While partial or split liver transplantation is associated with higher risks, outcomes in experienced centers can be comparable to those of full graft transplantation for carefully selected recipients [16]. Thus, the imbalance in the number of partial or split grafts in our cohort needs to be mentioned but is unlikely to have influenced the results and, if anything, might have favored better outcomes in the dual-HOPE group. In addition, we performed a sensitivity analysis by excluding recipients of partial and Re-liver transplantations from the original Propensity Score-matched cohort, and there were still no significant differences regarding one-year patient survival, one-year graft survival, NAS, and HLS between the mono- and dual-HOPE group.

The absence of significant differences in survival outcomes or NAS rates may also be attributed to the heterogeneity of the study population, which included both ECD and non-ECD donors. The complex interplay of additional risk factors, such as donor comorbidities and graft steatosis, in ECD grafts likely overshadows the potential benefits of dual-HOPE. These findings suggest that dual-HOPE might have a more pronounced impact in specific subgroups of particularly vulnerable grafts. Future studies with larger cohorts are warranted to explore these subgroup effects, especially for marginal donor organs, which may benefit more substantially from advanced perfusion strategies. However, for this indication, normothermic machine perfusion could also play a complementary role, particularly for high-risk organs [15]. Normothermic perfusion enables functional graft assessment prior to transplantation, which is critical for determining organ viability. While liver viability can be assessed by mitochondria-derived flavin mononucleotide values in perfusate during hypothermic perfusion [17], normothermic perfusion allows viability assessment with physiological biomarkers like lactate clearance, pH maintenance, or bile production [18–20]. Under normothermic conditions, dual vascular perfusion may be more important, as oxygen consumption is significantly higher, and inadequate hepatic artery flow could exacerbate the risk of complications like cholangiopathy. Additionally, the multifactorial nature of post-transplant complications necessitates a comprehensive approach beyond perfusion strategies alone.

One limitation of our study is the absence of donation after circulatory death (DCD) grafts, as no DCD program exists in Germany. DCD organs are a primary target for dynamic preservation techniques in other countries [21, 22]. Thus, the lack of DCD grafts limits the generalizability of our findings, particularly in settings where DCD transplantation is more common. Expanding future studies to include DCD organs would provide a broader understanding of dual-HOPE’s potential utility.

In conclusion, dual-HOPE did not demonstrate a significant advantage over mono-HOPE regarding patient survival rates, graft survival rates, or post-transplant NAS for DBD liver grafts. Expanding the study population and incorporating long-term follow-up could better elucidate the potential benefit of dual vascular perfusion, especially in ECD liver transplantation. Such insights would be pivotal in refining machine perfusion strategies to optimize graft preservation and patient outcomes.

## Data Availability

The raw data supporting the conclusions of this article will be made available by the authors, without undue reservation.

## Glossary

AIH: Autoimmune hepatitis
BC: Biliary complications
COD: Cause of death
DBD: Donation after brain death
DCD: Donation after cardiac death
DRI: Donor risk index
DGF: Delayed graft function
ECD: Extended criteria donor
ERC: Endoscopic retrograde cholangiography
ET-DRI: Eurotransplant-Donor Risk Index
GGT: Gamma-Glutamyl-Transferase
HCC: Hepatocellular Carcinoma
HLS: Hospital length of stay
HOPE: Hypothermic oxygenated machine perfusion
HTK: Histidine-tryptophan-ketoglutarate solution
ICU: Intensive Care Unit
IRI: Ischemia-reperfusion injury
labMELD: lab Model for End-stage Liver Disease
LC: Liver cirrhosis
LF: Liver Failure
LiTx: Liver transplantation
MELD: Model for End-stage Liver Disease
MRCP: Magnetic resonance cholangiopancreatography
MRI: Magnetic resonance imaging
NAFLD: Non-alcoholic fatty liver disease
NAS: Non-anastomotic biliary strictures
PBC: Primary biliary cholangitis
PRS: Post-reperfusion syndrome
PSC: Primary sclerosing cholangitis
PSM: Propensity Score Matching
PTC: Percutaneous cholangiography
ReLiTx: Re-liver transplantation
ReReLiTx: Re-Re-liver transplantation
ROS: Reactive oxygen species
SCC: Secondary sclerosing cholangitis
SCS: Static cold storage
SMD: Standardized mean differences
UW-MPS: University of Wisconsin machine perfusion solution
